# Kidney cancer epidemiology in a French Afro-descendant population: high prevalence of papillary renal cell carcinoma

**DOI:** 10.1007/s00345-026-06260-0

**Published:** 2026-02-10

**Authors:** Cécile Freymann, Anne-Claire Aurore, Vincent Lethongsavarn, Pascal Blanchet, Emilien Seizilles de Mazancourt, Laurent Brureau, Kevin Kaulanjan

**Affiliations:** 1 Department of Urology, CHU de Guadeloupe, University of Antilles, Pointe-à-Pitre, France; 2 Department of Pathology, CHU de Guadeloupe, University of Antilles, Pointe-à-Pitre, France; 3 IRSET (Institut de Recherche en Santé, Environnement et Travail) - UMR_S 1085, Pointe-à-Pitre, Guadeloupe France; 4 Department of Urology, Hopital Saint-Louis, Paris, France

**Keywords:** Urology, Kidney cancer, Epidemiology, Papillary renal cell carcinoma, African descent

## Abstract

**Purpose:**

Ethnic disparities in renal cell carcinoma (RCC) subtypes have been well documented in the United States (US), but no comparable data exist for Afro-descendant populations living within a European healthcare system. In the US, several studies have shown a significantly higher incidence of papillary renal cell carcinoma among populations of African descent. The aim of this study is to analyze the epidemiology of kidney cancer in a French Caribbean population, mainly of African descent.

**Methods:**

This retrospective study included all patients who underwent total or partial nephrectomy, or renal biopsy at the University Hospital of Guadeloupe between January 2016 and October 2022. Data on clinical features, tumor characteristics, biology, histology and survival were collected. Statistical analyses were performed using StatView version 5.0 software.

**Results:**

A total of 126 patients had been included between January 2016 and October 2022. The median age at diagnosis was 66.0 years [IQR: 57.2–71.8]. Overall, 70% of patients had arterial hypertension, 12% were on dialysis and 5.6% had a history of kidney transplantation. Clear cell renal cell carcinoma accounted for 43% of cases and Papillary renal cell carcinoma for 36% of cases.

**Conclusion:**

These findings highlight unique epidemiological patterns in a French Afro-descendant populations and emphasize the need for molecular and clinical research focused on papillary RCC. These findings suggest potential environmental and genetic influences in the development of PRCC.

## Introduction

Renal cell carcinoma (RCC) is the seventh most common cancer in France, with 14,700 new cases reported in 2020. It is the third most frequent urological cancer, following prostate and bladder cancer, and ranks as the 11th leading cause of cancer-related mortality across both genders. In France, the incidence of RCC has been steadily increasing since the 1990s, while the mortality rate has remained relatively stable [1, 2]

RCC comprises various histological subtypes, with clear cell renal cell carcinoma (ccRCC) being the most prevalent (70–80%), followed by papillary renal cell carcinoma (PRCC) (10–15%) and chromophobe renal cell carcinoma (3–5%) [3]. Recent updates in histological classification highlight the clinical and pathological complexity and heterogeneity of kidney cancer [4].

The geographic and ethnic distribution of RCC histological subtypes is heterogeneous, with studies from the United States reporting a higher incidence of papillary renal cell carcinoma (PRCC) among individuals of African descent [5–8]. In France, the population is divided between mainland France, predominantly of Caucasian descent, and overseas territories such as the French West Indies, where the population is primarily of African descent [9]. Both territories benefit from a uniform, universal healthcare system that provides equal access to care [10]. However, no epidemiological studies have evaluated RCC subtype distribution in Afro-descendant populations within Europe, representing a major gap in the international literature.

Given the heterogeneity of RCC and the demographic diversity of the French population, this study aims to investigate potential disparities in the epidemiology and histology of kidney cancer, with a particular focus on the French West Indian population. We hypothesize a higher prevalence of PRCC in this population, consistent with patterns observed in other Afro-descendant groups worldwide.

## Materials and methods

### Study population

In this retrospective study conducted at the University Hospital of Guadeloupe between January 2016 and October 2022, we included patients who underwent total or partial nephrectomy or renal biopsy. Patients with non-tumoral renal pathologies, non-renal cell tumors, or secondary renal location from another primary site were excluded. Guadeloupe, an overseas French department and archipelago located in the Caribbean, has a population of approximately 394 000, of whom about 80% are of African descent. The University Hospital of Guadeloupe is the only tertiary referral center for kidney cancer on the island, which minimizes selection bias and ensures near-exhaustive case capture for this population. Access to healthcare is equivalent to that of mainland France.

### Data collection

**Fig. 1:**
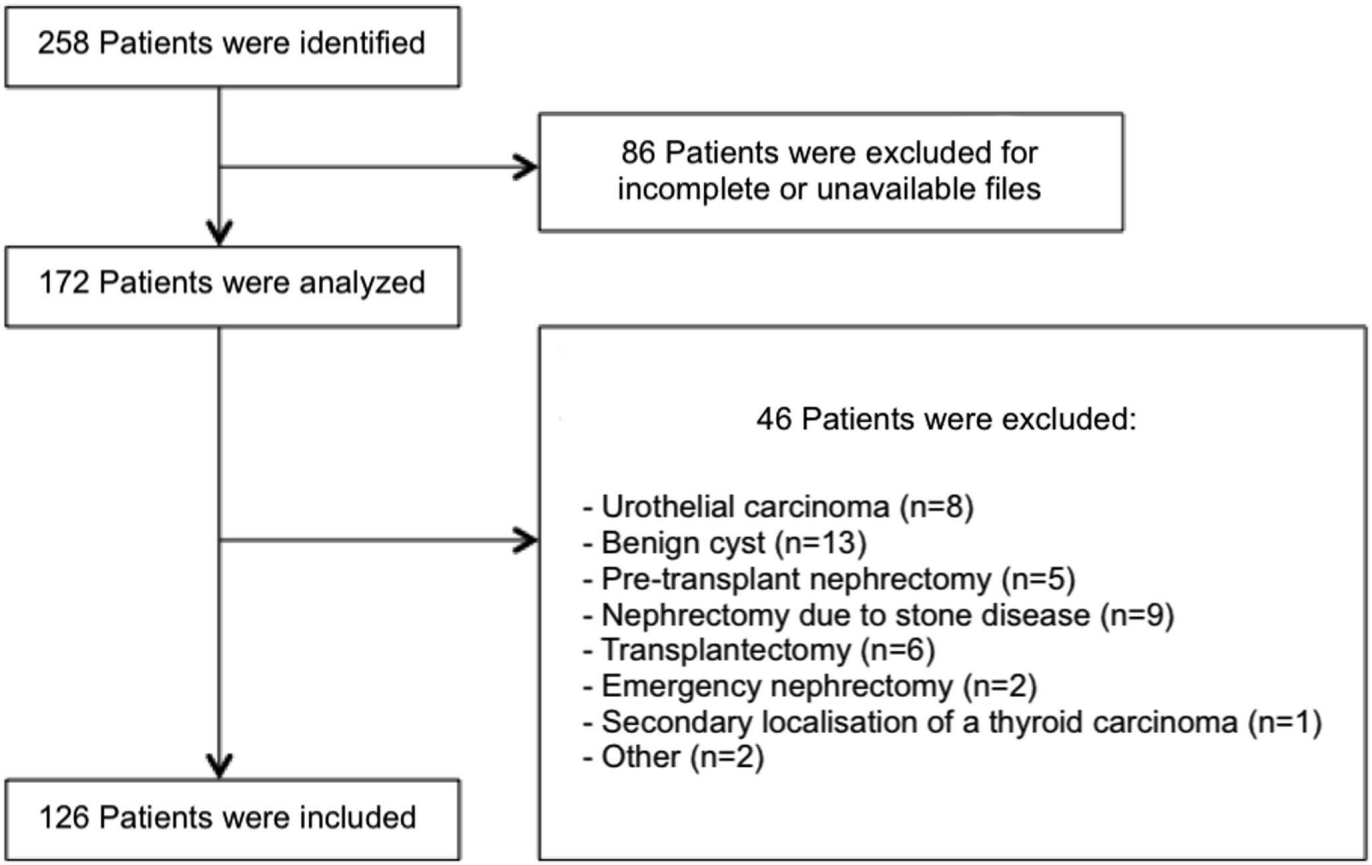
Flow chart

Patients who underwent total or partial nephrectomy or renal biopsy were identified using the DIAMIC^®^ pathology laboratory software and the ADICAP codes for renal surgical specimens (HUR) and renal biopsies (PUR). Clinical and paraclinical data were collected using the hospital’s software and included demographic and clinical characteristics such as age at diagnosis, known risk factors (body mass index [BMI], hypertension, smoking, chronic kidney disease [CKD], history of dialysis or kidney transplantation), presenting symptoms, tumor features (solid or cystic nature, size), locoregional extension (lymph node involvement, venous thrombosis), distant metastasis, surgical technique (partial or total nephrectomy), prior biopsy and the use of adjuvant medical treatment. Biological data included blood counts, renal function parameters, and estimated glomerular filtration rate (GFR). Histological data encompassed tumor subtype, ISUP (International Society of Urological Pathology) nucleolar grade, pathological T stage (pT), and surgical margin status. All histological specimens were centrally reviewed by two dedicated uropathologists to ensure diagnostic consistency. Missing data were handled through complete-case analysis. As the University Hospital of Guadeloupe is the sole referral center on the island, selection bias was minimized. Follow-up data included information on recurrence and long-term survival (Fig. 1).

### Ethics approval statement

Given the retrospective design and anonymized dataset, consent to participate did not apply. The study was approved by the Ethics Committee of the Pointe-à-Pitre University Hospital and registered with the French Data Protection Authority (CNIL) under registration number 2229605.

### Statistical analysis

Descriptive statistics were presented as medians with interquartile ranges (IQR) for continuous variables, and as percentages for categorical variables. Recurrence-free survival was defined as the time from treatment initiation to the diagnosis of clinical or radiological recurrence. Due to the limited number of deaths (*n* = 11), overall survival analysis could not be reliably performed. Statistical analyses were conducted using StatView software, version 5.0. All tests were two-sided, and a p-value of < 0.05 was considered statistically significant. 

## Results

We included 126 patients between January 2016 and October 2022. The median age at diagnosis was 66.0 years [IQR: 57.2–71.8], with 66.5 years [58.0–71.0] for men and 63.0 years [56.8–72.0] for women. Approximately two-thirds of the cohort were men (*n* = 90, 71.4%), while the remaining one-third were women (*n* = 36, 28.6%). More than 90% of patients presented with good overall health status, as indicated by a World Health Organization (WHO) performance status of 0 or 1. Partial nephrectomy was performed in 60% of cases, while total nephrectomy was performed in 38%. Laparoscopic procedures accounted for 88% of surgeries, with 48% of these being robot-assisted.

In terms of established risk factors for kidney cancer, the median body mass index (BMI) was 27.4 kg/m² [IQR: 24.7–30.1], with a median of 26.3 kg/m² [24.0–29.8] for men and 29.3 kg/m² [26.4–32.0] for women. Across all genders, 70% of patients had arterial hypertension. The median glomerular filtration rate (GFR) was 70.0 mL/min [IQR: 48.0–89.2]. Additionally, 12% of patients were undergoing dialysis, and 5.6% had a history of kidney transplantation.

In 75% of cases, the diagnosis was incidental. Renal biopsy was performed in 15% of patients. Regarding tumor characteristics, the median tumor size was 4.15 cm [IQR: 3.00–6.00]; 81% of tumors were solid and 19% cystic. Tumor thrombus involving the renal vein or vena cava was observed in 7.2% of cases. Regional lymph node involvement was found in 7.2% of patients, and distant metastases in 8.7%. Most tumors were localized, with 47% classified as stage T1a and 31% as stage T1b (Table 1).

**Table 1:** Population characteristics

	Male (*n* = 90)	Female (*n* = 36)	Total (*n* = 126)	*P*-value
Median age (years) [IQR]	66.5 [58.0–71.0]	63.0 [56.8–72.0]	66.0 [57.2–71.8]	0.59
PS (n; %)				
0	40 (70)	10 (45)	50 (63)	0.10
1	15 (26)	10 (45)	25 (32)	
2	2 (3.5)	2 (9.1)	4 (5.1)	
BMI (kg/m^2^) [IQR]	26.3 [24.0-29.8]	29.3 [26.4–32.0]	27.4 [24.7–30.1]	< 0.01
Smoking status (n ; %)	11 (14)	1 (3.4)	12 (11)	0.17
Hypertension (n ; %)	61 (68)	27 (75)	88 (70)	0.42
Creatinine (µmol/l) [IQR]	101 [83.0-128]	83.0 [65.2–132]	100 [80.0-130]	0.02
GFR (ml/min) [IQR]	70.0 [55.0–86.0]	68.0 [40.5–92.5]	70.0 [48.0-89.2]	0.74
Dialysis (n ; %)	10 (11)	5 (14)	15 (12)	0.76
Renal transplant (n ; %)	5 (5.6)	2 (5.6)	7 (5.6)	1.00
Diagnosis (n ; %)				
Incidental	71 (79)	24 (67)	95 (75)	0.15
Symptomatic	19 (21)	12 (33)	31 (25)	
Tumor size (cm) [IQR]	4.00 [3.00-6.30]	4.50 [3.65-6.00]	4.15 [3.00–6.00]	0.56
Tumor aspect (n ; %)				
Solid	73 (81)	29 (81)	102 (81)	0.94
Cystic	17 (19)	7 (19)	24 (19)	
Thrombus (n ; %)	6 (6.7)	3 (8.3)	9 (7.2)	0.72
N (n ; %)	6 (6.7)	3 (8.3)	9 (7.2)	0.72
M (n ; %)	9 (10)	2 (5.6)	11 (8.7)	0.72
Biopsy (n ; %)	14 (16)	5 (14)	19 (15)	0.81
Type of surgery (n ; %)				
PN	60 (67)	16 (44)	76 (60)	0.02
TN	28 (31)	20 (56)	48 (38)	
Procedure (n ; %)				
Laparotomy	12 (14)	2 (5.6)	14 (11)	0.26
Laparoscopy	32 (36)	18 (50)	50 (40)	
Robot-assisted laparoscopy	44 (50)	16 (44)	60 (48)	
pT Stage (n ; %)				
T1a	38 (51)	13 (37)	51 (47)	0.64
T1b	22 (30)	12 (34)	34 (31)	
T2a	3 (4.1)	4 (11)	7 (6.4)	
T2b	2 (2.7)	1 (2.9)	3 (2.8)	
T3a	6 (8.1)	4 (11)	10 (9.2)	
T3b	1 (1.4)	0 (0)	1 (0.92)	
T3c	1 (1.4)	0 (0)	1 (0.92)	
T4	1 (1.4)	1 (2.9)	2 (1.8)	

*IQR* Interquartile range;* PS* WHO Performance status;* BMI* Body mass index;* GFR* Glomerular filtration rate;* N* Regional lymph nodes;* M* Metastasis;* PN* Partial nephrectomy;* TN* Total nephrectomy

**Fig. 2:**
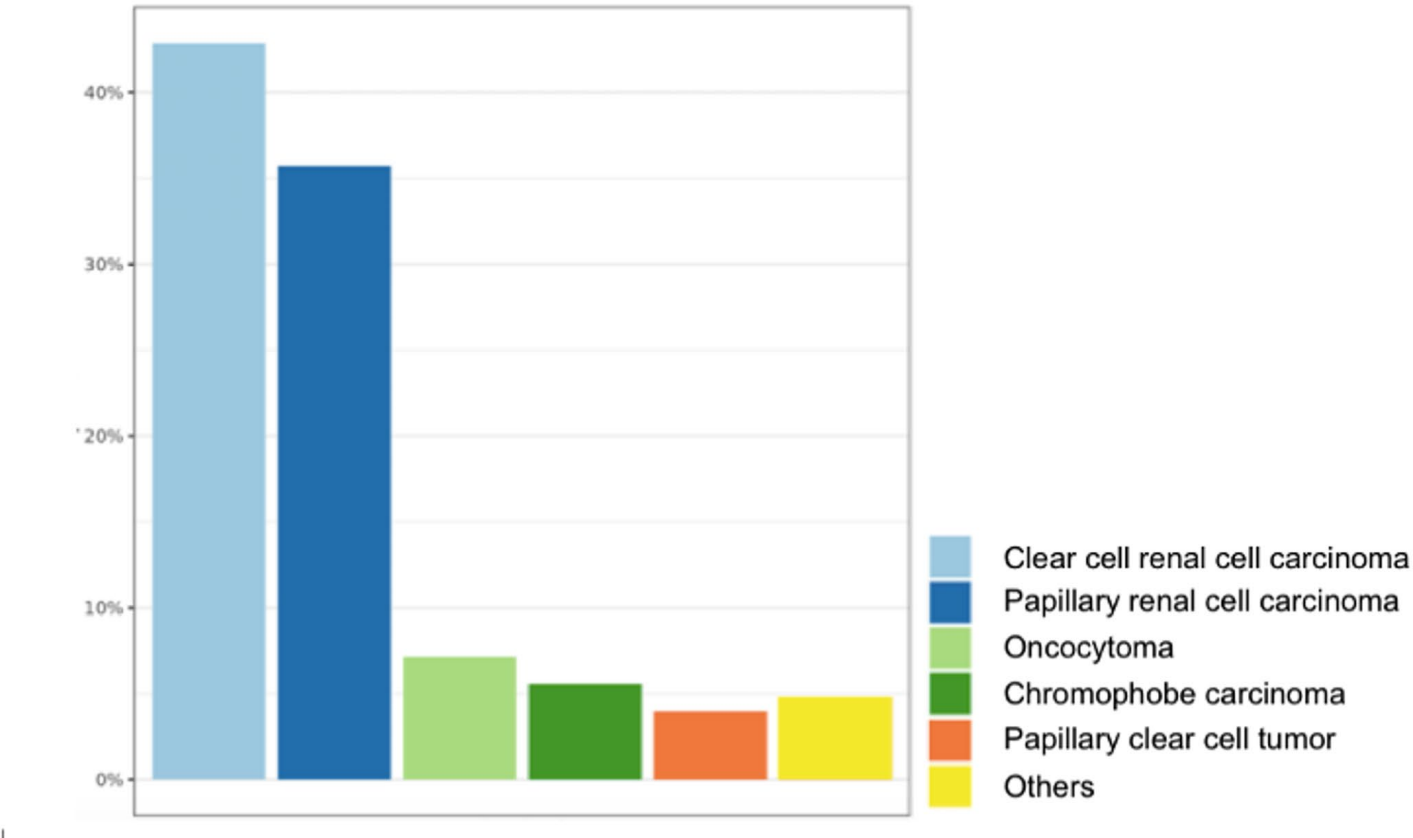
Histological subtype frequency in Guadeloupe

In terms of histological subtypes, clear cell renal cell carcinoma (ccRCC) was the most common and accounted for 43% of cases. The proportion of PRCC observed in our cohort (36%) is more than double the expected rate in Western populations (typically 10–15%). Other tumor types included chromophobe RCC in 5.6% of cases and oncocytomas in 7.2%. Papillary clear cell tumors were identified in 4% of cases (Table 2; Fig. 2). Additional rare subtypes included renal carcinomas with *TFE3* rearrangement and renal carcinomas with *TFEB* rearrangement.

**Table 2:** Histological subtype distribution of patients treated in Guadeloupe

	Number	Percentage (%)
Clear cell renal cell carcinoma	54	43
Papillary renal cell carcinoma	45	36
Chromophobe carcinoma	7	5.6
Oncocytoma	9	7.2
Papillary clear cell tumor	5	4
Others	6	4.8

The analysis of population and tumor characteristics according to the two main histological subtypes, clear cell RCC (ccRCC) and papillary RCC (PRCC), revealed a lower median glomerular filtration rate (GFR) in the PRCC group compared to the ccRCC group (65.0 mL/min [IQR: 31.0–79.0] vs. 72.0 mL/min [IQR: 57.0–90.8], *p* < 0.05). The PRCC group also showed a significantly higher proportion of patients undergoing dialysis (20% vs. 5.6%, *p* < 0.05) and with a history of kidney transplantation (13% vs. 1.9%, *p* < 0.05). Rates of hypertension and median BMI [IQR] were comparable between the two groups. The median tumor size was significantly larger in the ccRCC group (5.00 cm [3.50–7.15] vs. 4.00 cm [2.70–5.60], *p* < 0.05). No significant differences were observed between groups in terms of distant metastasis or tumor stage (Table 3).

**Table 3:** Distribution of characteristics according to the two main histological types

	ccRCC (*n* = 54)	PRCC (*n* = 45)	*P*-value
Sex (*n*; %)			
Female	18 (33)	10 (22)	0.22
Male	36 (67)	35 (78)	
Median age (years) [IQR]	41.7 [38.1–45.9]	36.2 [33.0-44.1]	0.81
BMI (kg/m^2^) [IQR]	29.1 [25.0-30.3]	26.6 [23.7–29.0]	0.09
Smoking status (n ; %)	9 (20)	3 (8.6)	0.17
Hypertension (n ; %)	37 (69)	34 (76)	0.44
Creatinine (µmol/l) [IQR]	93.0 [75.2–121]	112 [91.5; 185]	0.013
GFR (ml/min) [IQR]	72.0 [57.0-90.8]	65.0 [31.0; 79.0]	0.033
Dialysis (n ; %)	3 (5.6)	9 (20)	0.028
Renal transplant (n ; %)	1 (1.9)	6 (13)	0.044
Tumor size (cm) [IQR]	5.00 [3.50–7.15]	4.00 [2.70–5.60]	0.043
Thrombus (n ; %)	5 (9.4)	3 (6.7)	0.72
N (n ; %)	3 (5.7)	4 (8.9)	0.70
M (n ; %)	6 (11)	3 (6.7)	0.50
ISUP (n ; %)			
1	3 (5.7)	3 (6.8)	0.72
2	29 (55)	22 (50)	
3	16 (30)	17 (39)	
4	5 (9.4)	2 (4.5)	
pT Stage (n ; %)			
T1a	19 (37)	24 (56)	0.13
T1b	20 (39)	11 (26)	
T2a	3 (5.9)	2 (4.7)	
T2b	1 (2)	2 (4.7)	
T3a	7 (14)	1 (2.3)	
T3b	0 (0)	1 (2.3)	
T3c	0 (0)	1 (2.3)	
T4	1 (2)	1 (2.3)	
Death (n ; %)	3 (5.6)	7 (16)	0.18

*ccRCC* Clear cell renal cell carcinoma;* PRCC* Papillary renal cell carcinoma;* BMI* Body mass index;* GFR* Glomerular filtration rate;* N* Regional lymph nodes;* M* Metastasis;* ISUP* International Society of Urological Pathology

## Discussion

This study provides the first European data showing a markedly increased prevalence of papillary RCC in an Afro-descendant population living within a uniform healthcare system. In fact, clear cell renal cell carcinoma (ccRCC) emerged as the most common histological subtype, (43% of cases) followed by papillary renal cell carcinoma (PRCC) with 36% of cases. Although this study was not designed as a comparative analysis, the incidence of PRCC is notably higher than in mainland France, where PRCC typically accounts for only 10–15% of renal tumors [3]. This suggests that the frequency of PRCC in the Guadeloupean population is approximately threefold higher. The homogeneity of African ancestry in the Guadeloupean population offers a unique opportunity to explore ethnic disparities in RCC without major socioeconomic confounders. These findings highlight a distinct distribution of renal cancer subtypes between two French populations with comparable healthcare access but differing ethno-geographical backgrounds. These findings are consistent with previous scientific investigations. For example, Olshan et al., analyzing data from over 52,000 patients in the SEER (Surveillance, Epidemiology, and End Results) database across 18 U.S. populations, reported that patients of African descent were four times more likely to present with PRCC compared to Caucasian patients (23% vs. 9%, respectively) [7]. Similarly, Sankin et al. found PRCC in 47.9% of Afro-descendant patients, compared to just 10.3% in Caucasian patients [6]. Lipworth et al. also noted a threefold higher proportion of PRCC among patients of African descent relative to Caucasians (35.7% vs. 13.8%) [5]. More recently, Lu et al., based on data from the French Kidney Cancer Research Network (UroCCR), reported ethnic disparities in the epidemiology of renal cell carcinoma among Afro-descendant populations. These findings corroborate the results of our study, further emphasizing the influence of ethnic background on the distribution of histological subtypes in kidney cancer [11].

Comparison of clinical characteristics between the two main histological subtypes revealed that patients with PRCC had a significantly lower median glomerular filtration rate (GFR) [IQR: 65.0 mL/min, 31.0–79.0] compared to those with ccRCC [IQR: 72.0 mL/min, 57.0–90.8] (*p* < 0.05). Additionally, the proportion of patients undergoing dialysis was significantly higher in the PRCC group (20% vs. 5.6% for ccRCC, *p* < 0.05), as was the prevalence of prior kidney transplantation (13% vs. 1.9% for ccRCC, *p* < 0.05) (Table 3). The higher prevalence of certain risk factors such as hypertension and chronic kidney disease (CKD) in the Afro-Caribbean population may partially explain the overrepresentation of PRCC in this cohort, consistent with evidence linking chronic renal injury to PRCC tumorigenesis [12, 13]. Several retrospective studies have highlighted an association between CKD and an increased likelihood of PRCC histology [14]. For instance, Klatte et al. reported a notably higher frequency of PRCC (43% vs. 19%) in native kidney tumors of renal transplant patients [15].

Another plausible explanation lies in genetic predispositions among individuals of African descent. Although abnormalities in karyotype and alterations in the MET signaling pathway have been identified in PRCC, the molecular underpinnings remain poorly understood [3]. Further genomic studies are needed to clarify these mechanisms and their potential link to racial and ethnic disparities in RCC subtypes. A study using The Cancer Genome Atlas (TCGA) database found genomic differences between Afro-descendant and Caucasian patients with PRCC, implicating immune response signaling pathways and the VEGF signaling pathway. Additionally, overexpression of a gene associated with the WNT signaling pathway, implicated in carcinogenesis, was observed in Afro-descendant patients [16]. In the context of targeted therapies and personalized medicine, gaining a deeper understanding of the genetic abnormalities driving PRCC is essential for guiding therapeutic decisions and improving patient outcomes. While recent studies in the management of metastatic ccRCC evaluating combinations of immunotherapy and antiangiogenic agents have led to new treatment guidelines, similar robust evidence for metastatic PRCC remains lacking. Given the limited efficacy of current systemic therapies for metastatic PRCC, identifying high-risk populations is essential for optimizing surveillance and guiding future therapeutic research. Most therapeutic trials to date have predominantly enrolled Caucasian populations, underscoring the urgent need for additional epidemiological, clinical, and molecular research. The inclusion of Afro-descendant cohorts in studies of metastatic kidney cancer, particularly PRCC, is essential to ensure equitable and effective therapeutic strategies [17].

This study has several limitations. First, the study relied on the WHO 2016 classification rather than the updated 2022 version. However, this limitation does not affect the identification of major RCC subtypes, including PRCC. Secondly, the retrospective and single-center design of the study, as well as the relatively small sample size, also represent some limitations. Although all patients were managed within a universal healthcare system, the absence of individual-level socioeconomic data constitutes a major limitation of this study, as socioeconomic and environmental factors may still influence disease presentation and outcomes. Furthermore, the limited number of patients and the relatively short follow-up period prevented reliable assessment of survival outcomes. Finally, because the collection of individual ethnic data is legally prohibited in France, ethnicity could not be assessed through self-identification or genetic ancestry, and population-level demographic data were used instead, which may have introduced misclassification bias. Despite these limitations, our work provides novel and necessary baseline data for renal cell carcinoma in an afro descendant population, which may serve as a foundation for future comparative, multicentric, or hypothesis-driven studies.

## Conclusion

This study identifies a significantly increased prevalence of papillary RCC in a French Afro-descendant population, suggesting potential genetic and renal impairment–related mechanisms driving this disproportionate prevalence. These findings underscore the need for dedicated molecular studies and improved representation of Afro-descendant populations in RCC clinical trials.

## Data Availability

The clinical datasets generated and analyzed during the present study are not publicly available due to patient confidentiality regulations and restrictions imposed by the French Data Protection Authority (CNIL). However, de-identified data may be made available from the corresponding author upon reasonable request and subject to approval by the Ethics Committee of the University Hospital of Guadeloupe. No publicly archived datasets were used in the study.
